# Do Younger Patients with Muscle-Invasive Bladder Cancer have Better Outcomes?

**DOI:** 10.3390/jcm8091459

**Published:** 2019-09-13

**Authors:** Florian Janisch, Hang Yu, Malte W. Vetterlein, Roland Dahlem, Oliver Engel, Margit Fisch, Shahrokh F. Shariat, Armin Soave, Michael Rink

**Affiliations:** 1 Department of Urology, Medical University of Hamburg, Martinistraße 52, 20246 Hamburg, Germany; drfjanisch@gmail.com (F.J.); yuhang.seu@outlook.com (H.Y.); malte.vetterlein@googlemail.com (M.W.V.); r.dahlem@uke.de (R.D.); o.engel@uke.de (O.E.); m.fisch@uke.de (M.F.); armin.soave@googlemail.com (A.S.); 2 Department of Urology, Medical University of Vienna, Währinger Gürtel 18-20, 1090 Vienna, Austria; sfshariat@gmail.com; 3 Institute for Urology and Reproductive Health, Sechenov University, Bolshaya Pirogovskaya str. 2-4, 119991 Moscow, Russia; 4 Department of Urology, Weill Cornell Medical School, 1300 York Avenue, New York, NY 10065, USA; 5 Department of Urology, University of Texas Southwestern Medical Center, 5323 Harry Hines Blvd, Dallas, TX 75390, USA; 6 Karl Landsteiner Institute of Urology and Andrology, Franziskanergasse 4, a 3100 St. Poelten, Austria; 7 Department of Urology, Second Faculty of Medicine, Charles University, Ovocný trh 5, Prague 1-116 36, Czech Republic

**Keywords:** bladder cancer, age, urothelial carcinoma, radical cystectomy, outcome, survival

## Abstract

Urothelial cancer of the bladder (UCB) is usually a disease of the elderly. The influence of age on oncological outcomes remains controversial. This study aims to investigate the impact of age on UCB outcomes in Europe focusing particularly on young and very young patients. We collected data of 669 UCB patients treated with RC at our tertiary care center. We used various categorical stratifications as well as continuous age to investigate the association of age and tumor biology as well as endpoints with descriptive statistics and Cox regression. The median age was 67 years and the mean follow-up was 52 months. Eight patients (1.2%) were ≤40 years old and 39 patients (5.8%) were aged 41–50 years, respectively. In multivariable analysis, higher continuous age and age above the median were independent predictors for disease recurrence, and cancer-specific and overall mortality (all *p*-values ≤ 0.018). In addition, patients with age in the oldest tertile group had inferior cancer-specific and overall survival rates compared to their younger counterparts. Young (40–50 years) and very young (≤40 years) patients had reduced hazards for all endpoints, which, however, were not statistically significant. Age remains an independent determinant for survival after RC. Young adults did, however, not have superior outcomes in our analyses. Quality of life and complications are endpoints that need further evaluation in patients undergoing RC.

## 1. Introduction

With an incidence of over 80,000 new cases and over 17,000 deaths estimated to occur in 2019 in the United States alone, urothelial cancer of the bladder (UCB) is the second leading genitourinary malignancy and a potentially lethal disease [1]. Compared with other malignancies, UCB is usually a disease of the elderly with a peak incidence among those in their 70s [2,3]. In fact, in general there is an increasing life expectancy in the US and Europe and in consequence, a potential further rise in UCB diagnoses is expected in the next few decades [4,5]. Ageing trends are of major scientific and clinical importance in any cancer including UCB, as the optimal management has great impact for each individuum and the public health system in general, especially in an expensive disease as UCB [6].

Despite the overwhelming incidence in elderly patients, UCB does also occur in a non-negligible number of young patients [3]. While the development of UCB in the elderly has been suggested to be driven by a cumulative lifetime exposure to environmentally, occupationally, or individually acquired carcinogens (e.g., smoking) [7,8,9], the factors for UCB in young patients remain rather inconclusive. Not only the diagnosis of UCB, particularly the need of RC with all its negative effects on quality of life has a significant impact on the psyche and more, especially in the younger. RC is more frequently offered in younger patients, due to their longer life expectancy, lower frailty resulting in lower adverse events and the superiority in survival outcomes of early compared to delayed RC [10]. Recent reports suggest superior UCB-specific outcomes in young and adolescent patients (15–39 years) [11]. 

The impact of patient age on oncological outcomes remains controversial and regional variabilities may be present that need to be considered in patient counselling and treatment planning. The aim of this study was to evaluate the impact of age on UCB outcomes after RC in a consecutive cohort of European patients, particularly focusing on the young and very young. We hypothesized that younger patients may have better oncologic outcomes as their disease may be earlier in their natural history and different as it may not have a large mutational burden.

## 2. Material and Methods

### 2.1. Patient Population

We retrospectively reviewed the medical records of 789 consecutive patients treated with RC and bilateral pelvic lymphadenectomy for UCB between 1996 and 2011 at our institution. Guideline adherent indications for RC were muscle invasive UCB or recurrent Ta, T1, or carcinoma in situ (CIS) refractory to transurethral resection of the bladder (TURB) with or without intravesical chemo- or immunotherapy. As neoadjuvant chemotherapy may be more frequently administered in younger patients, implementing an inherent bias of natural UCB history in age analyses, these patients were excluded upfront (*n* = 8). Moreover, 75 patients were excluded because of missing variables or follow-up, 30 patients with RC for non-malignant indication for RC, and seven patients with advanced, bladder infiltrating prostate cancer. In total, 669 patients remained for analyses. Overall, 147 patients (20.0%) received adjuvant chemotherapy (95% platinum-based) at the clinicians’ discretion in accordance with the guidelines at the time. The study was approved by the local ethics committee.

### 2.2. Follow-Up Regimen

Follow-up strategy has been previously reported in detail [12,13]. In brief, patients were generally seen every three to four months for the first year after surgery, every six months from the second to fifth year, and annually thereafter. Follow-up included a history, physical examination and serum chemistry evaluation. Diagnostic imaging of the abdomen including the urinary tract and chest radiography were performed at least annually or when clinically indicated. Additional radiographic evaluations were performed when clinically indicated. 

### 2.3. Statistical Analysis

Statistical analyses included demographic data on patients’ age, ethnicity, gender, ASA status, pathologic tumor stage and grade, concomitant CIS, lymph node status, margin status, lymphovascular invasion, and adjuvant chemotherapy, respectively.

The co-primary endpoints were recurrence-free survival (RFS), cancer-specific survival (CSS), and overall survival (OS), respectively. Disease recurrence was defined as local failure in the operative site, regional lymph nodes, or distant metastasis. Upper tract urothelial carcinoma was considered a metachronous tumor and not disease recurrence. Patients who did not experience disease recurrence were censored at time of last follow-up for recurrence-free survival analysis. Cancer-specific mortality was defined as death from UCB. The cause of death was determined by the treating physician, by chart review corroborated by death certificates, or by death certificates alone [14]. Perioperative mortality (i.e., death within 30 days of surgery) was censored at time of death for UCB-specific survival analyses.

Age was analyzed as a continuous variable, with a cut-off at median and tertiles, and using the cut-offs of 50 years. (dichotomized) and ≤40, 41–50, and >50 years (three categories), respectively. The different analytic approaches were used to optimally approach the definition of young age. There is no clear determination for UCB patients treated with RC in the urologic literature defining a patient as ‘young’ or ‘very young’. However, there is a consensus among oncological experts that patients <50 years. are usually defined as ‘young’ and patients <40 years. defined as ‘very young’ [15]. Using median and tertiles as cut-off, we investigated the effects of age in our study population with homogenous sample distributions. Utilization of the dichotomized cut-off of 50 years. was based on previous reports that indicated superior survival outcomes in patients <50 years. The tri-categorical analyses uses cut-offs of <40 years and <50 years following predefined ranges indicated by the NCI in 2006 [15]. In addition, study results indicate significant outcome differences, suggesting these strata represent an ideal standard [11]. 

The Kolmogorov–Smirnov test was used to assess the normal distribution of variables. The Fisher’s exact test and the chi-square test were used to evaluate the association between categorical variables. Differences in variables with a continuous distribution across categories were assessed using the Mann–Whitney U test (two categories) and Kruskal–Wallis test (three and more categories). Actuarial method was used to estimate RFS, CSS, and OS probabilities and the differences were assessed with the log rank test. Kaplan–Meier estimates were used to graphically display survival functions. Univariable and multivariable Cox regression models addressed time-to-event endpoint analyses. In all models, proportional hazards assumptions were systematically verified using the Grambsch–Therneau residual-based test. Multicollinearity was assessed with the variance inflation factor to test for possible confounding between relevant covariates. All reported *p*-values were two-sided, and statistical significance was set at *p* < 0.05. All statistical tests were performed with IBM SPSS Statistics 25 (IBM Corp., Armonk, NY, USA).

## 3. Results

### 3.1. Association of Age with Clinical–Pathological Characteristics

The median age of the study cohort was 67 years (interquartile range [IQR]: 59; 73), and 520 (78%) of the patients were male. In total, 622 patients (93.0%) were older than 50 years. Of those being <50 years, 39 patients (5.8%) were aged 41–50 years and 8 patients (1.2%) were younger than 40 years. Tertiles for age were ≤59 years (first tertile), 60–72 years (second tertile), and ≥73 years (third tertile), respectively. The descriptive clinicopathologic characteristics of the study cohort are presented in Table 1. 

Comparing patients under and over 50, older patients presented with a significantly higher ASA score (*p* < 0.001). ASA scores increased significantly from patients ≤40 years to patients aged 41–50 and to ≥50 years (*p* < 0.001). There were no statistically significant differences in any other clinical–pathological variables irrespective of the stratification used. 

### 3.2. Association of Age with Disease Recurrence and Survival Outcomes

The median follow-up was 52 months (IQR: 17; 78). During the follow-up period, 192 patients (32.4%) experienced disease recurrence, 175 patients (28.0%) died of UCB, and 257 patients (42.1%) died of any cause. The actuarial recurrence-free survival estimates at 2- and 5-years after RC were 64 ± 2% and 59 ± 2%, respectively. The actuarial cancer-specific survival estimates at 2- and 5-years after RC were 71 ± 2% and 61 ± 3%, respectively. The actuarial overall-specific survival estimates at 2- and 5-years after RC were 62 ± 2% and 50 ± 2%, respectively. 

**Figure 1:**
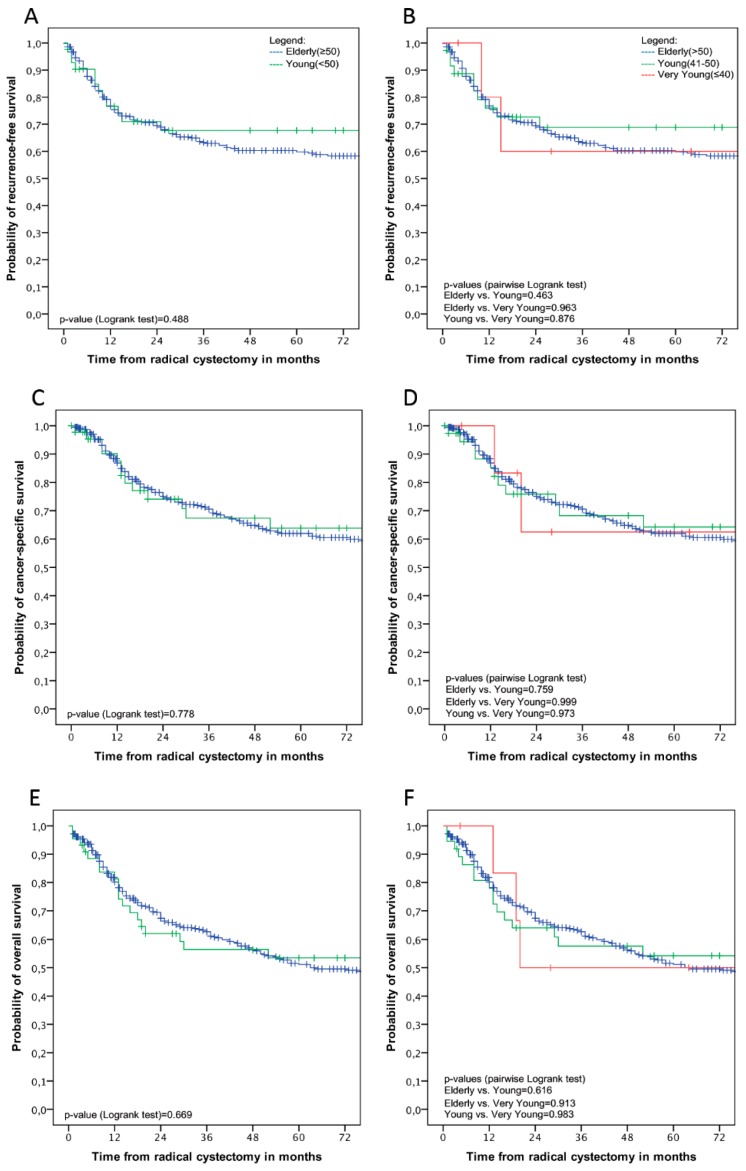
Kaplan–Meier estimates of stratified age groups of elderly (≥50 years.) and young patients (<50 years.) (**A**,**C**,**E**), and stratified in three age groups of elderly (>50 years.) young (41–50 years.) and very young (≤40 years.) patients (**B**,**D**,**F**) for recurrence-free, cancer-specific, and overall survival, respectively.

In the Kaplan–Meier analyses, no statistically significant difference was observed in recurrence-free survival (*p* = 0.49; Figure 1A), cancer-specific survival (*p* = 0.78; Figure 1C), and overall survival (*p* = 0.67; Figure 1E) between patients younger than 50 years, and those 50 and above. In categorical age group analyses, there was also no statistically significant difference in recurrence-free survival, cancer-specific survival and overall survival (*p* > 0.05 for all; Figure 1B,D,F) between patients 50 years and above, those between 41–50 years and those 40 and younger. 

### 3.3. Risk Factor Analyses for Disease Recurrence and Survival Outcomes

All variables tested on multicollinearity had an VIF in the range of 1.1–2.9, indicating that no multicollinearity is present between factors included in the cox regression model. The results of univariable Cox regression analyses for different age stratifications are presented in Table 2. Higher continuous age was significantly associated with inferior recurrence-free (Hazard ratio (HR): 1.017; 95%CI: 1.002–1.032; *p* = 0.029), cancer-specific (HR: 1.023; 95%CI: 1.007–1.039; *p* = 0.005), and overall survival (HR: 1.030; 95%CI: 1.016–1.044; *p* < 0.001). In addition, patients older than the median (all *p* ≤ 0.01) and patients in the highest age tertile compared to patients in the second tertile (all *p* ≤ 0.008) were significantly associated with inferior outcomes for all three endpoints. Analyses according to all age categories (i.e., ≤50 vs >50; ≤40 vs. 41–50 vs. >50, tertiles) revealed that patients in higher age categories were not associated with a higher risk for all endpoints (all *p* > 0.05). 

The results of multivariable Cox regression analyses that adjusted for standard UCB clinic-pathological parameters (Table 3) showed that higher continuous age (RFS HR: 1.019; *p* = 0.018; CSS HR: 1.025; *p* = 0.004, and OS HR: 1.030; *p* < 0.001), age above the median of our cohort (RFS HR: 1.472; *p* = 0.014; CSS HR: 1.553; *p* = 0.008; and OS HR: 1.596; *p* = 0.001) and age in the third tertile (≥73 years) compared to the second age tertile (60–72 years) (RFS HR: 1.862; *p* = 0.005; CSS HR: 2.085; *p* = 0.001; OS HR: 2.256; *p* < 0.001) were all independently associated with worse outcomes for all three endpoints. In addition, CSS and OS of patients in the third tertile were also inferior compared to the outcomes of patients in the first age tertile (≤59 years) (CSS HR: 1.545; *p* ≤ 0.034; OS HR 1.728; *p* = 0.002).

## 4. Discussion

We found that young patients did not present with more favorable tumor biological features compared to their older counterparts. In addition, we did not find significantly superior survival outcomes for all three endpoints in favor of young patients. Therefore, we reject our hypothesis that young UCB patients with MIBC have better outcomes post-RC than the normal MIBC patient. This is in contrast to previous studies that reported better oncological outcomes in younger UCB populations [11,16,17]. Differences in results between our study and previous reports may be explained by different definition of young age, diverse race/ethnicity or distinct socioeconomic status, etc. [18]. Indeed, we found that higher continuous age and other strata defining patients as elderly were independently associated with inferior survival outcomes. Thus, while one end of the age spectrum does not better, the other end of the spectrum seems to have worse oncologic outcomes. Undeniably, this underscores the validity of our UCB cohort as these findings are in line with those of large, multicentric UCB series [19,20].

The relationship between age and prognosis of UCB remains controversial. In fact, there is no clear definition when a UCB patient is defined to be very young, young, old, or very old. A recent large US study reported that adolescents and young UCB patients (ages 15–39) [11] had superior cancer-specific and overall survival compared to their older counterparts. In another study, patients were stratified using a cut-off of 50 years with superior cancer-specific and overall survival in the younger group [16]. In our study we, therefore, used variable age stratifications and cut-offs to reflect the most comprehensive picture on the impact of age across a wide spectrum of definitions and results. Indeed, using this comprehensive approach, we only found differences in categorized outcome analyses when using the median age or age tertiles of our cohort. Of importance, the median age in our cohort was 67 years and the upper age tertile included patients above 73 years. Thus, despite depicting a statistical significance compared to ‘younger patients’ in our cohort, our data did not demonstrate superior outcomes in those patients usually defined to be young or very young.

From the biological rationale it intuitively seems reasonable that older patients may experience inferior outcomes. With increasing age, exposure to several environmental, occupational, and individually amenable (e.g., smoking) stressors accumulate over time [7,21,22]. In addition, especially elderly men often experience obstructive lower urinary tract symptoms with incomplete bladder drainage. In consequence, the potentially prolonged contact time to carcinogens excreted in the urine may induce accumulation of cellular events that can lead to neoplastic transformation and subsequently UCB development [20]. Moreover, younger patients tend to be healthier in general, with mostly good immunity and nutrition status, as well as fewer co-morbidities to tolerate the complications of cancers or treatment.

From a clinical perspective, our results are important, as recent studies found very young UCB patients to have a lower hazard of cancer-specific mortality compared to their older counterparts [11], suggesting that organ-sparing approaches may be a viable option in young patients [23,24,25]. Especially the prospect of incontinence, impotence and/or infertility due to radical cystectomy may lead to delay of therapy or a switch of strategy to a bladder sparing, multimodal approach. However, younger patients are more reluctant to undergo necessary diagnostics and treatments or comply with strict follow-up schedules, possibly affecting outcomes [26]. In agreement with our results, other investigators also found either no difference [27] or even worse outcomes, due to a higher rate of metastases [28], in patients ≤40 years. A potential reason for the disparity in findings of various studies including ours may be due to difference in ethnicities, regional varieties in treatment, different socioeconomic backgrounds, and other factors that we could not all adjust for in our analyses. However, despite our findings not providing a final answer on the influence of a very young age on UCB outcomes treated with RC, our results do generate hypotheses that warrant further investigation of this association in larger, multi-institutional, ideally prospective studies. Indeed, our findings support the surgical approach of radical cystectomy also in young and very-young patients, as our findings underscore the aggressive nature of UCB and no age-group had superior survival outcomes. Certainly, the reconstruction which affects quality of life and perceived self-image should be adopted to patients’ preferences, and general and specific health factors [29].

Our study is not devoid of limitations. First and foremost, the retrospective and single-center nature may inevitably introduce some selection bias. Despite this being a large monocentric cohort of consecutive patients, the overall sample size is still limited, especially in the subgroup of very young patients. Nevertheless, we feel that our study still provides a representative insight on age-dependent prognostic outcomes for Europe. All patients in our study were Caucasians, which should be considered since ethnicity may influence survival in UCB [30,31]. We were unable to collect and adjust analyses for several predisposing risk factors of UCB, including smoking, occupational exposure, family history, insurance status, immunity or nutrition status, adjuvant therapies, or socio-economic factors that also may influence tumor biology or outcomes [22,32,33]. In addition, laboratory and molecular data was not available. However, from the clinical perspective, the latter information is usually not available in daily routine for patient counselling. Age in general does represent a competing risk for death particularly since older patients have a greater frailty and UCB patients often harbor important comorbidities [34,35,36]. However, due to sample size limitations, we were unable to perform competing-risk analyses. Consequently, a contemporary, European multicenter approach would be warranted to shed further insight on this relevant topic. 

## 5. Conclusions

In conclusion, we found that young age at time of MIBC diagnosis does not result in better outcomes compared to typical age after RC. Higher age, however, remains an important prognostic factor for cancer-related endpoints in UCB and thus needs to be incorporated in therapeutic considerations. Radical cystectomy remains standard treatment for patients with muscle-invasive bladder cancer independent of age. Further studies to assess the differential effect of RC and the different types of urinary diversion on the health-related quality of life and metabolic consequences across age are necessary. 

## Figures and Tables

**Table 1:** Descriptive characteristics stratified by dichotomy age groups of 669 UCB patients treated with radical cystectomy

	All	Young (≤50)		Elderly (≥51)	*p*-Value	*p*-Value
		≤40	41–50	≥51	≤50 vs. ≥51	≤40 vs. 41–50 vs. ≥51
Patients, *n*	669	8	39	622	-	-
Gender (%)					0.85	0.98
Male	520 (77.7)	6 (75.0)	30 (76.9)	484 (77.8)		
Female	149 (22.3)	2 (25.0)	9 (23.1)	138 (22.2)		
ASA (%)					<0.001	<0.001
1	52 (7.8)	4 (50.0)	7 (18.0)	41 (6.6)		
2	386 (57.7)	3 (37.5)	27 (69.2)	356 (57.2)		
3	225 (33.6)	1 (12.5)	3 (7.7)	221 (35.5)		
4	6 (0.9)	0 (0)	2 (5.1)	4 (0.7)		
Pathological Tumor Stage (%)					0.92	0.34
pT0	73 (10.9)	3 (37.5)	4 (10.3)	66 (10.6)		
pTa	26 (3.9)	0 (0)	1 (2.6)	25 (4.0)		
pTis	66 (9.9)	0 (0)	3 (7.7)	63 (10.1)		
pT1	74 (11.1)	1 (12.5)	4 (10.3)	69 (11.1)		
pT2	132 (19.7)	0 (0)	10 (25.6)	122 (19.6)		
pT3	182 (27.2)	4 (50.0)	8 (20.5)	170 (27.3)		
pT4	116 (17.3)	0 (0)	9 (23.1)	107 (17.2)		
Pathological Tumor Grade (%)					0.58	0.42
No grading (pT0)	73 (10.9)	3 (37.5)	4 (10.3)	66 (10.6)		
G2	65 (9.7)	1 (12.5)	6 (15.4)	58 (9.3)		
G3	531 (79.4)	4 (50.0)	29 (74.3)	498 (80.1)		
Concomitant carcinoma in situ (%)					0.051	0.12
Absent	424 (63.4)	7 (87.5)	29 (74.4)	388 (62.4)		
Present	245 (36.6)	1 (12.5)	10 (25.6)	234 (37.6)		
Lymph node status (%)					0.91	0.58
pN0	479 (71.6)	7 (87.5)	27 (69.2)	445 (71.5)		
pN+	190 (28.4)	1 (12.5)	12 (30.8)	177 (28.5)		
Margin status (%)					0.94	0.99
R0	586 (87.6)	7 (87.5)	34 (87.2)	545 (87.6)		
R+	83 (12.4)	1 (12.5)	5 (12.8)	77 (12.4)		
Lymphovascular invasion (%)					0.52	0.62
L0	455 (68.0)	6 (75)	24 (61.5)	425 (68.3)		
L1	214 (32.0)	2 (25)	15 (38.5)	197 (31.7)		
Adjuvant Chemotherapy (%)					0.089	0.21
No	522 (78.0)	6 (75.0)	26 (66.7)	490 (78.8)		
Yes	147 (22.0)	2 (25.0)	13 (33.3)	132 (21.2)		

**Table 2:** Univariable cox regression analysis of variable age stratifications predicting recurrence-free survival, cancer-specific survival and overall survival of 669 patients with UCB treated with radical cystectomy

Age Stratifications	RFS			CSS			OS		
	HR	95%CI	*p*-Value	HR	95%CI	*p*-Value	HR	95%CI	*p*-Value
Continuous age	1.017	1.002–1.032	0.029	1.023	1.007–1.039	0.005	1.030	1.016–1.044	<0.001
Median Age	1.454	1.095–1.932	0.010	1.550	1.150–2.088	0.004	1.663	1.299–2.129	<0.001
Age ≤50 vs. >50	1.227	0.684–2.202	0.49	1.084	0.616–1.909	0.78	1.107	0.693–1.767	0.67
Age (three categories)									
≤40 vs. >50	0.818	0.179–3.733	0.80	0.910	0.202–4.111	0.90	0.832	0.242–2.858	0.77
41–50 vs. >50	1.035	0.257–4.170	0.96	1.001	0.248–4.038	0.99	0.946	0.303–2.955	0.92
Age (Tertiles)									
first vs. third tertile	1.242	0.861–1.793	0.25	1.364	0.926–2.008	0.12	1.576	1.137–2.185	0.006
second vs. third tertile	1.699	1.151–2.507	0.008	1.931	1.278–2.917	0.002	2.194	1.546–3.112	<0.001

Abbreviations: RFS = recurrence free survival; CSS = cancer specific survival; OS = overall survival; HR = hazard ratio; CI = confidence interval; UCB = urothelial carcinoma of the bladder.

**Table 3:** Multivariable cox regression analysis of the effect of age on predicting recurrence-free survival, cancer-specific survival and overall survival of 669 patients with UCB and treated with radical cystectomy

Age Stratifications	RFS			CSS			OS		
	HR	95%CI	*p*-Value	HR	95%CI	*p*-Value	HR	95%CI	*p*-Value
Continuous Age	1.019	1.003–1.035	0.018	1.025	1.008–1.042	0.004	1.030	1.016–1.045	0.000
Median age	1.472	1.081–2.005	0.014	1.553	1.124–2.146	0.008	1.596	1.225–2.080	0.001
Age (Tertiles)									
first vs. third tertile	1.339	0.914–1.962	0.13	1.545	1.033–2.312	0.034	1.728	1.230–2.428	0.002
second vs. third tertile	1.862	1.211–2.864	0.005	2.085	1.328–3.276	0.001	2.256	1.541–3.304	<0.001

All multivariable analyses were adjusted for the following co-variables: gender, ASA score, pathological tumor stage, pathological tumor grade, concomitant carcinoma in situ, lymph node status, margin status, lymphovascular invasion, and adjuvant chemotherapy. Abbreviations: RFS = recurrence free survival; CSS = cancer specific survival; OS = overall survival; HR = hazard ratio; CI = confidence interval; UCB = urothelial carcinoma of the bladder.
